# Dissecting Oxidative Stress and Organismic Response to a Temperature Gradient in the Midge *Chironomus riparius*

**DOI:** 10.1002/ece3.72625

**Published:** 2025-12-11

**Authors:** Burak Bulut, Maximilian Geiss, Markus Bernard, Halina Binde Doria, Barbara Feldmeyer, Markus Pfenninger

**Affiliations:** ^1^ Department of Molecular Ecology Senckenberg Biodiversity and Climate Research Centre Frankfurt am Main Germany; ^2^ Institute for Molecular and Organismic Evolution Johannes Gutenberg University Mainz Germany; ^3^ LOEWE Centre of Translational Biodiversity Genomics Senckenberg Biodiversity and Climate Research Centre Frankfurt am Main Germany

**Keywords:** antioxidant response, climate change, insect, oxidative stress, temperature stress

## Abstract

Oxidative stress, caused by reactive oxygen species (ROS), poses a major challenge for organisms facing temperature fluctuations. This study provides the first direct in vivo measurements of ROS production together with transcriptome analysis of oxidative stress genes across a broad range of ecologically relevant temperatures in insect larvae of the dipteran midge *Chironomus riparius* . We observed a U‐shaped pattern of oxidative stress, with minimal ROS levels within an optimal thermal window (12°C–18°C) and significantly elevated stress at both cold and warm extremes. Crucially, our findings reveal distinct underlying molecular mechanisms for ROS generation at these extremes: at low temperatures, predominantly ROS produced is of the superoxide group, linked to hypoxia‐induced hemoglobin autoxidation. Conversely, at high temperatures, the hydrogen peroxide group dominates, associated with increased metabolic rate and heat stress signaling pathways. Transcriptomic analysis shows that *C. riparius* 's antioxidant defense system adapts accordingly, selectively upregulating mechanisms to counteract the specific dominant ROS type at different temperatures. This mechanistically differentiated oxidative stress at different temperatures and the modulated organismic response reflect the ecological niche and evolution of *C. riparius* .

## Graphical abstract

This study of the insect *Chironomus riparius* reveals a U‐shaped pattern of oxidative stress, with the lowest stress levels at optimal temperatures. Our findings show that different types of ROS are generated at thermal extremes, driven by distinct molecular mechanisms—hypoxia at low temperatures and metabolic rate at high temperatures. These results suggest a mechanistically differentiated response to temperature that reflects the species' ecological niche.

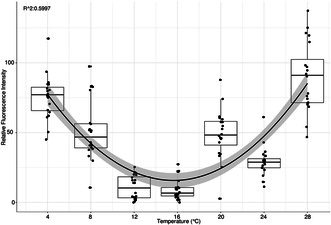

## 1 Introduction

In their natural habitats, most organisms are subjected to a range of temperatures throughout their lifespan. Temperature can vary across multiple temporal and spatial scales, ranging from gradual changes across seasons overlain by multiday weather periods and rapid fluctuations over the course of a single day, as well as spatial thermal heterogeneity within the habitat. These fluctuations are especially pronounced in temperate regions, where seasonal shifts in temperature can be substantial, requiring organisms to cope with prolonged periods of suboptimal or stressful conditions (Heise et al. 2007). Given that temperature influences the rate of all chemical reactions, each temperature represents a distinct environmental condition that ultimately affects organismal fitness (Kiekebusch et al. 2024). Deviations from the optimal temperature range that the organism is adapted to may therefore result in increased stress, diminished fitness, and in the extreme, mortality (Gasparrini et al. 2015).

A primary consequence of temperature‐related stress is the disruption of cellular homeostasis, which elicits a complex, integrated stress response. This response is multifaceted, involving an array of protective mechanisms. A key component is the upregulation of *heat shock proteins* (HSPs), which function as molecular chaperones to refold denatured proteins and maintain cellular integrity under thermal duress (Hagymasi et al. 2022). Concurrently, organisms often engage in membrane remodeling, adjusting the lipid composition of cellular membranes to maintain optimal fluidity and function across different temperatures, a process known as homeoviscous adaptation (Los and Leusenko 2025). Another reason for temperature‐related stress is the production of reactive oxygen species (ROS) (Szechyńska‐Hebda et al. 2022). ROS are highly reactive molecules that necessarily occur in every living cell. The majority of ROS generated within cells is produced by mitochondria as an unavoidable by‐product of the electron transport chain, primarily by complexes I and III (Hernansanz‐Agustín and Enríquez 2021; Pfenninger and Foucault 2020). The actual ROS composition produced depends strongly on the stress‐inducing conditions and the organism's condition and may thus vary widely (Das and Roychoudhury 2014). While the broader cellular stress response is critical for survival, this study focuses on providing a mechanistic understanding of the oxidative stress component of thermotolerance.

To prevent the damage caused by ROS, cells have evolved sophisticated antioxidant defense systems that include both nonenzymatic and enzymatic components. The non‐enzymatic antioxidant system, whose basic concept is to produce bait substances that are oxidized by ROS instead of essential cell components (e.g., vitamin C, glutathione, etc.; Chinta and Andersen 2008; Liu et al. 2020). On the other hand, the enzymatic antioxidant system produces diverse enzymes to directly turn ROS into less harmful substances. This system encompasses superoxide dismutase, catalase, glutathione peroxidase, thioredoxin, peroxiredoxin, and glutathione transferase (Jomova et al. 2024). Antioxidant enzymes are distributed throughout various cellular compartments to counteract ROS wherever they occur (Nwachukwu et al. 2021). However, ROS molecules are not only harmful; they are essential for a multitude of cellular processes, including signaling and the defense against xenobiotic agents. As a result, cells attempt to maintain a delicate equilibrium between ROS production and removal by the antioxidant systems (Mackova et al. 2024; Sikder et al. 2025).

If ROS molecules are not swiftly scavenged, they cause widespread damage to macromolecules, which translates directly into fitness costs. The oxidation of metabolic enzymes (proteins) can impair energetic efficiency, while damage to cell membranes (lipids) can disrupt cellular transport and signaling, directly impacting growth and survival (Ntawubizi and Mukamuhirwa 2024). Crucially, oxidative damage to DNA not only compromises somatic cell function but can also introduce mutations into the germline, a link that is a powerful mechanism for evolutionary change (Dowling and Simmons 2009). Therefore, understanding how organisms manage oxidative stress is fundamental to explaining variation in key life history traits such as metabolic efficiency, growth rate, and transgenerational genomic integrity.

Studies of temperature‐induced ROS effects have predominantly focused on plant systems, resulting in a substantial body of literature on the mechanisms and impacts of oxidative stress (Devireddy et al. 2021). The few prior studies in animals examining temperature‐related oxidative stress employed indirect methods, such as measuring enzyme activity and/or gene expression of antioxidative genes (Yang et al. 2024; Zhang et al. 2015). Moreover, previous studies investigating ROS production have predominantly focused on high (Farahani et al. 2020; Zhang et al. 2014) or low (El‐Saadi et al. 2023; Lebenzon et al. 2023) temperature conditions, and only a few have examined both thermal extremes within the same framework (Cui et al. 2011; Yang et al. 2010).

To our knowledge, no study has yet systematically measured ROS production in vivo across a continuous, ecologically relevant temperature gradient in an insect model. This represents a significant research gap, particularly because physiological responses to temperature are rarely linear. Thermal performance curves typically show that stress is minimized within an optimal window and overproportionally increases at both colder and warmer extremes (Pörtner et al. 2006). In *C. riparius* specifically, this U‐shaped pattern has been documented for spontaneous mutation rates (Waldvogel and Pfenninger 2021). Based on these theoretical grounds and empirical observations, we hypothesize that overall ROS levels will also exhibit a U‐shaped pattern, with minimal production within an optimal thermal window and significantly elevated levels at both cold and warm extremes.

This study aims to test this hypothesis by providing the first direct in vivo measurements of ROS production across a broad thermal gradient in *C. riparius* , alongside a transcriptomic analysis of its antioxidant defense system. Characterizing this relationship is critical for understanding the evolutionary implications of thermal stress. Variation among individuals in their ability to manage ROS can create fitness differentials, providing the raw material for natural selection to act upon (Huchzermeyer et al. 2022). Therefore, by dissecting the mechanistic underpinnings of temperature‐induced oxidative stress, we are investigating a key physiological process that can determine a population's capacity to adapt to rising average temperatures and increased thermal extremes, or conversely, highlight its vulnerability in the face of climate change (Said and Nassar 2022).

## 2 Material and Method

### 2.1 Test Animal and Experimental Design

We selected the nonbiting midge *Chironomus riparius* for this study due to a combination of its ecological, practical, and physiological attributes. Ecologically, it is a widespread freshwater insect in temperate regions, central to benthic food webs, where its aquatic larvae are naturally exposed to significant daily and seasonal temperature fluctuations (Oppold et al. 2016). Practically, it is a well‐established model organism in ecotoxicology and evolutionary biology, with standardized rearing protocols and extensive genomic resources available (Foucault et al. 2019; Pettrich et al. 2024). Physiologically, *C. riparius* possesses a unique hemoglobin‐based respiratory system that makes it highly tolerant of hypoxia but also particularly susceptible to specific forms of oxidative stress linked to hemoglobin autoxidation, providing a useful system for investigating these mechanisms.

As a widespread freshwater insect, *C. riparius* inhabits rivers, lakes, and ponds across diverse climatic zones, including Hessen, Germany, where our lab population originates. In this region, temperature fluctuates seasonally and geographically, with an annual mean of approximately 9.74°C (Waldvogel and Pfenninger 2021). Seasonal averages provide a more nuanced perspective: during spring and summer (May to September), the main reproductive period temperatures typically range from 13°C to 18°C, while occasional summer heatwaves or warmer microhabitats may push temperatures higher. In contrast, winter temperatures (November to February) often drop below 5°C, sometimes approaching 2°C (Oppold et al. 2016).

All experimental animals originated from a long‐term laboratory culture established in 2015 (Oppold et al. 2016) with individuals from the Hasselbach stream in Hessen, Germany (50°12′N, 8°37′E). The stock population is maintained under controlled conditions at 20°C on a 16:8 light:dark photoperiod. Cultures are housed in plastic tray aquaria containing a 1.5 cm layer of fine sand (> 0.2 μm) and are supplied with constant aeration. Larvae are fed *ad libitum* with commercial fish food (TetraMin). To minimize inbreeding and prevent significant laboratory adaptation, the culture is maintained with a large breeding population size (> 500 individuals per generation) and is refreshed every 2 years with new wild‐caught individuals from the source population. All larvae used in the experiments were reared under these standard conditions until the start of the thermal treatments.

For all experiments, L3‐stage larvae were selected from this stock culture, due to their established physiological robustness and active metabolic state, making them highly responsive to thermal stressors. This stage avoids the practical challenges associated with handling smaller L1 and L2 larvae and the confounding physiological and transcriptomic changes related to beginning metamorphosis in L4 larvae (Eberhardt et al. 2025). A total of 20 L3‐stage larvae were collected from this laboratory culture for each treatment and placed in 24‐well plates. The plates were filled with 2.5 mL of the medium described in detail in (Foucault et al. 2018; Oppold et al. 2016).

We used a relatively recently developed method to measure ROS levels in living organisms (Kang et al. 2013). The use of cell‐permeable fluorogenic probes allows for the measurement of ROS‐related fluorescence intensity within the cells of living organisms. Two different reagents were used to identify the different ROS under different temperatures: CellROX Orange (Thermo Fisher cat. no. C10443) and CellROX Red reagents (Thermo Fisher cat. no. C10422). In the reduced state, the reagents are nonfluorescent. However, following oxidation by ROS, they exhibit fluorogenic signals at 545/565 nm for CellROX Orange and 640/665 nm excitation/emission wavelengths for CellROX Red. CellROX Orange is capable of detecting five distinct ROS, including hydrogen peroxide, hydroxyl radical, nitric oxide, peroxynitrite anion, and superoxide anion. In contrast, CellROX Red only detects two ROS, namely the hydroxyl radical and superoxide anion, which are also part of CellROX Orange. The difference between the CellROX Red and the CellROX Orange signal thus gives a hint at the relative proportion of the two ROS groups produced. Even though these substances are present in excess in the cells, they cannot scavenge all ROS molecules upon their emergence; the method nevertheless allows an accurate relative estimation of ROS production (Wu et al. 2019).

Single L3 larvae were placed in a single well of a 24 well‐plate (Greiner Bio‐One 662,160) in a climate chamber with a 16:8 light/dark cycle and a light intensity of 550 lx, without active aeration. The relatively large surface‐to‐volume ratio of the 2.5 mL medium in the wells allowed for sufficient passive oxygen diffusion to support larval respiration over the 24‐h experimental period, minimizing systemic hypoxia as a confounding factor. The 24‐h exposure duration was specifically chosen to capture the acute oxidative stress response to temperature shifts, reflecting ecologically relevant rapid fluctuations experienced by *C. riparius* larvae in their natural habitats. Experiments were conducted under a 16:8 light/dark cycle to account for potential influences of circadian rhythm on physiological responses. The study employed seven distinct consecutive temperature regimes, encompassing 4°C, 8°C, 12°C, 16°C, 20°C, 24°C, and 28°C. In total, we measured 20 larvae per temperature treatment. Larvae were randomly selected from a continuously breeding population pool, and due to the asynchronous nature of the population, individuals likely originated from different females. Consequently, sampling on consecutive days is not expected to introduce systematic variation.

### 2.2 Reactive Oxygen Species Measurement

Following 24 h of treatment, the well plates were placed in a styrofoam box for transportation to the microscope to prevent any abrupt temperature change. ROS levels were estimated for a total of 20 larvae per temperature and CellROX dye type. Two larvae had to be excluded from further analysis because they died in the process (one from the 20°C treatment group and one from the 28°C treatment group for CellROX Orange).

A preliminary experiment with different concentrations of CellROX stock solution (0.5–2.5 μL per 2.5 mL of medium; results not shown) helped us to determine the final working concentrations for the main experiments. Each larva was stained with CellROX Orange at a final concentration of 1.5 μM (0.75 μL of 5 mM stock per 2.5 mL medium) and CellROX Red at a final concentration of 2.5 μM (1.25 μL of 5 mM stock per 2.5 mL medium). These concentrations were optimized to provide detectable signals with a non‐detrimental concentration.

ROS levels were quantified in live larvae using a ZEISS Axio Imager 2 microscope with 10× magnification. The images were captured using AxioVision Rel. (v. 4.8) with an HXP 120 C fluorescence lamp (Item Number: 423013‐9010‐000), with maximum light intensity and 1‐s exposure time. We furthermore employed the “43 HE” (BP 550/25 HE, FT 570 HE, BP 605/70 HE, Item Number 489043‐9901‐000) filter, which excites blue light at approximately 550 nm, transmitting emitted red fluorescence above 570 nm and filtering out the remaining blue excitation light, thus registering only red fluorescence around 605 nm. As the L3 larvae were too large for complete body imaging, fluorescence measurements were consistently performed in the first abdominal segment (Figure S2). This segment was selected due to its identifiability, accessibility, and suitability for consistent fluorescence measurement across all individuals.

The fluorescence field images were analyzed using the ImageJ Fiji software (version 2.15.0). Images were converted to 8‐bit grayscale from RGB color images to eliminate color differences, to be able to solely calculate light intensity. The CellROX Orange and CellROX Red dyes exhibited differential brightness under identical fluorescence illumination conditions. Consequently, distinct thresholds were applied to the respective reagent treatments. Because the maximum fluorescence light intensity was applied, consequently the background noise (background light) was elevated. Therefore, the auto threshold was set too high to lower the background noise. The mean values were taken as a measure of fluorescence intensity. It should be noted that the fluorescence intensity measured represents the relative amount of reagent that has entered the cell and undergone oxidation by binding to ROS, rather than an absolute quantification of ROS present within the cell. However, the optimized concentration of CellROX reagents used was sufficient to capture the ROS present within the cells across all conditions, ensuring that our measurements reflect true differences in ROS production. While direct quantification of dye uptake or its intrinsic temperature sensitivity was not performed, the optimized nonlethal concentrations of reagents were applied uniformly across all temperature treatments to ensure consistent and relative measurements of ROS dynamics in vivo.

### 2.3 Gene Expression Analysis

For gene expression analysis, a distinct experimental setup was employed compared to the acute ROS measurements, designed to capture transcriptional responses and adaptive changes over longer periods. A total of 45 L3 larvae were collected from the lab culture for each temperature treatment. The larvae were exposed to specific temperature treatments in glass bowls filled with sand and medium, as previously described (Foucault et al. 2019). Unlike the CellROX experiments, the temperatures used for transcriptomics were 5°C, 10°C, 15°C, 20°C, and 25°C. This difference in temperature ranges between the CellROX and transcriptomic experiments was due to logistical constraints and financial considerations inherent to each distinct experimental setup, though both ranges effectively cover ecologically relevant thermal conditions for *C. riparius* . Larvae were provided with aeration and permitted to feed at their discretion, which was essential for these longer‐term exposures (2–5 days) to allow for complete transcriptional responses and to account for temperature‐dependent developmental rates. As developmental time depends on temperature in this ectothermic organism, the duration of exposure varied accordingly: 2 days for 25°C, 3 days for 20°C, 4 days for 15°C and 10°C, and 5 days for 5°C.

For each temperature treatment, multiple replicates of larvae were utilized, with 12 individuals per 25°C, 15°C, and 10°C groups, and 11 individuals per 20°C and 5°C groups. For each individual larva, a separate RNA library was prepared. RNA was extracted using the Direct‐zol RNA Miniprep Kits (R2053; Zymo Research) according to the manufacturer's manual. Library preparation and sequencing were conducted at Novogene on a NovaSeq X Plus instrument.

### 2.4 RNA Data Analysis

The FASTQ files were aligned to the *C. riparius* reference genome v4 (Pettrich et al. 2024) which was pre‐indexed for efficient mapping. Alignment was performed using HISAT2 (v. 2.1.0; Kim et al. 2019). Subsequently, the resulting SAM files were sorted and converted to BAM format using Samtools (v. 1.20; Li et al. 2009). Finally, read counts for each gene per individual sample were quantified using FeatureCounts (version 2.0.6; Liao et al. 2014).

Raw counts were subsequently normalized using DESeq2 (v. 3.19; Love et al. 2014) with read counts < 10 in at least 4/5 of all tested temperatures were excluded to remove lowly expressed genes and reduce noise, resulting in the selection of 118 genes. In this study, we focused on ROS‐related genes and their specific reaction norms to different temperatures only. To this end, protein sequences of 134 ROS‐related proteins from the model organism *Drosophila melanogaster* were downloaded from UniProt on 30 June 2024. *Drosophila* was selected as a well‐annotated insect model, which is a dipteran and a close phylogenetic relative of *C. riparius* , making its ROS‐related protein set a highly relevant reference (Akinyemi et al. 2018; Bag and Mishra 2020; Yang et al. 2024). Subsequently, the *C. riparius* genome was queried against the downloaded protein database using BLASTx (v. 2.12.0; Camacho et al. 2009) to identify and extract the corresponding genes, including those related to mitochondrial ETC components. With this approach, nine previously annotated genes were discovered in the *C. riparius* genome and incorporated into the analysis. The *C. riparius* protein set was queried against the UniProtKB protein database using the InterProScan (v. 5.52) software to obtain the gene name.

### 2.5 Statistical Analysis

All statistical analyses and data visualizations were performed in R version 4.4.1 (“R: A Language and Environment for Statistical Computing | BibSonomy” n.d.).

### 2.6 Analysis of ROS Data

To test for a significant effect of temperature on overall ROS levels (Relative Fluorescence Intensity, RFI), we performed an Analysis of Variance (ANOVA). To test our hypothesis of a U‐shaped relationship between temperature and ROS, we fitted a second‐order polynomial regression model to the data. The fit of this model was compared to a linear model using the Akaike Information Criterion (AIC) to confirm that the polynomial model provided a better description of the data.

### 2.7 Differential Gene Expression Analysis

Differential gene expression analysis was performed to identify genes with significant expression changes between the temperature (15°C) and each of the other thermal treatments (5°C, 10°C, 20°C, and 25°C). The analysis was conducted in R, using the DESeq2‐normalized count data for the 118 selected ROS‐related genes as input. The limma package (v. 3.60.2) was employed for its robust statistical framework (Ritchie et al. 2015). Prior to modeling, the normalized expression values were log2‐transformed, with a pseudocount of 1 added to accommodate zero values. A linear model was then fitted for each gene, and empirical Bayes moderation was applied to increase statistical power. Contrasts were established to compare each temperature group against the 15°C, since it is close to the optimal temperature. *p* values were adjusted for multiple testing using the Benjamini‐Hochberg method to control the false discovery rate. The results were visualized as volcano plots using the ggplot2 package, where the *x*‐axis represents the log_2_ fold change and the *y*‐axis represents the negative log_10_ of the adjusted *p* value. Genes were colored according to their ROS target classification (superoxide, hydrogen peroxide, general ROS) to highlight the response patterns of different functional groups.

### 2.8 Principal Component Analysis (PCA)

To explore coordinated temperature‐dependent responses in the antioxidant system, we conducted a principal component analysis (PCA) exclusively on 23 genes known to be directly involved in ROS production or scavenging (Ryan et al. 2020). The goal was to identify dominant patterns of variation in gene expression across the temperature gradient and to uncover how antioxidant systems respond collectively under thermal stress. The normalized gene expression data for these antioxidant genes were imported into R and preprocessed by applying z‐score normalization. PCA was performed using the “prcomp()” function to capture the major axes of variation in antioxidant gene expression across temperature treatments. Gene loadings obtained from the PCA rotation matrix were combined with ROS target annotations, where the sign and magnitude of the loading indicate the direction and strength of a gene's contribution to the variance along a principal component, providing insight into its expression pattern across temperatures. Sample metadata, including temperature, were extracted from sample identifiers and merged with PCA scores. Additionally, a broken stick model was used to assess the significance of the variance explained by each principal component.

Visualization of the PCA results was carried out using the ggplot2 package (Wickham et al. 2019). A biplot was generated, displaying samples in the PC1–PC2 space with points colored by temperature and gene loadings represented as scaled arrows. Complementary boxplots were also created to compare the distribution of principal component scores across temperature treatments, with custom color scales applied for clarity.

## 3 Results

### 3.1 Temperature Dependent Stress Levels Across Temperatures Detected via ROS

**FIGURE 1:**
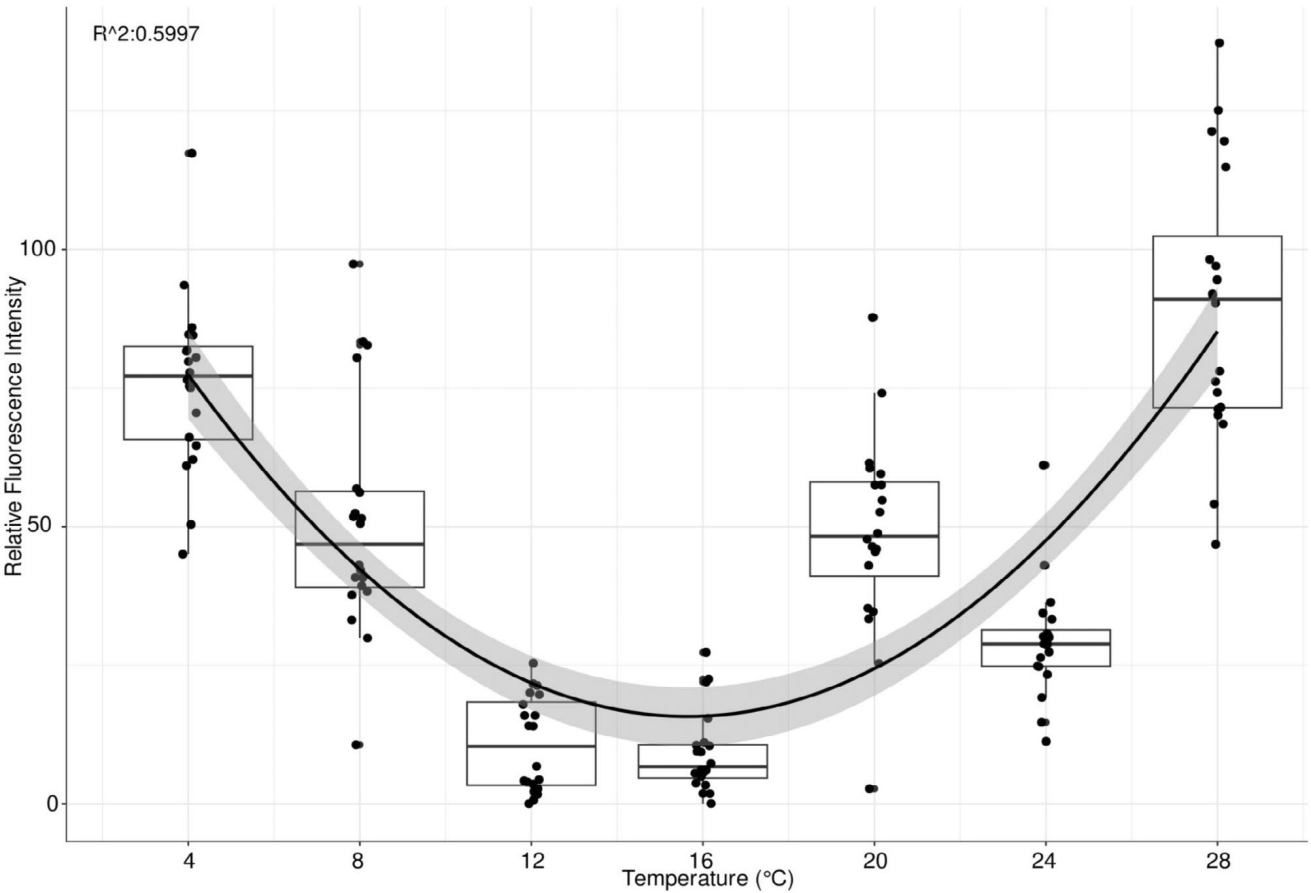
Boxplot depicting the relative fluorescence intensity (RFI) across different temperatures based on CellROX Orange measurements, which detects a broader spectrum of ROS. The distribution of RFI across temperatures fits a parabolic distribution (*R* ^2^ = 0.5997; *F*‐statistic: 105.1 on 2 and 137 DF, *p* value: < 2.2e‐16). The line shows the fitted regression with a 95% confidence interval (shaded area).

The stress levels of larvae as measured by the relative fluorescence intensity (RFI) of ROS exhibited a significant temperature dependence (ANOVA, *F*(6, 133) = 64.02, *p* < 0.001), indicating relative changes in oxidative activity across the thermal gradient. RFI was highest at the two temperature extremes and lowest between 12°C and 18°C, which indicates an optimal temperature range (Figure 1). Fitting a second‐order polynomial regression model to the data revealed a U‐shaped relationship between temperature and RFI (goodness of fit: *R* ^2^ = 0.6, *p* value < 2.2e‐16; formula: 44.91672 + 30.85863*x + 298.3746*x ^2^). The polynomial regression model fitted better to the data compared to the linear model, according to AIC values of 1250 and 1377, respectively.

### 3.2 Characterization of ROS Types Under Different Temperature Conditions

**FIGURE 2:**
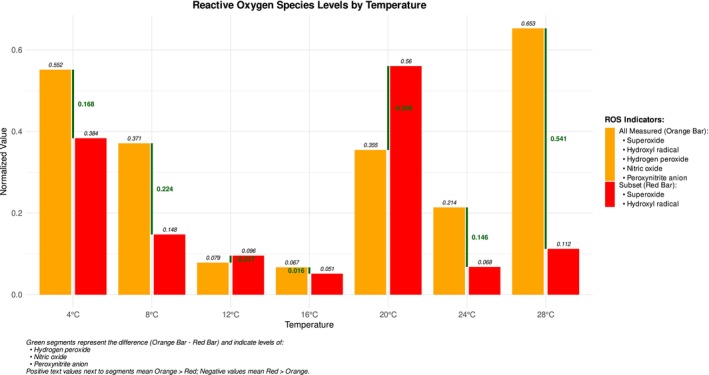
Normalized fluorescence intensity of ROS indicated by orange bars (total measured ROS by CellROX Orange) and red bars (subset of ROS by CellROX Red) at temperatures ranging from 4°C to 28°C. This figure illustrates the relative abundance and qualitative shifts of different ROS species across temperatures, inferred from the differential sensitivity of CellROX Orange and CellROX Red. Total measured ROS includes superoxide, hydroxyl radical, hydrogen peroxide, nitric oxide, and peroxynitrite anion. The subset of ROS consists of superoxide and hydroxyl radicals. The figure is based on normalized fluorescence intensity data, with orange bars representing total ROS pool and red bars representing the superoxide and hydroxyl radical subset. Green segments, with corresponding numerical values, illustrate the difference between total and subset ROS levels (CellROX Orange—CellROX Red), representing the combined contribution of hydrogen peroxide, nitric oxide, and peroxynitrite anion. For quantitative data and statistical validation of the overall temperature effect, refer to Figure 1.

When assessing the relative abundance of ROS species across temperatures, it becomes evident that the qualitative contribution of different ROS groups, inferred from the differential sensitivity of the CellROX probes, changes with temperature. At 4°C, the superoxide group (detected by CellROX Red) predominated, indicating a higher relative contribution of these species. In contrast, at 24°C and 28°C, there was an elevated level of the hydrogen peroxide group (inferred from the CellROX Orange signal, encompassing hydrogen peroxide, nitric oxide, and peroxynitrite), suggesting their increased relative contribution at warmer temperatures (Figure 2). At optimal temperatures, the overall amount of ROS was lower than at the extremes, and the superoxide group appeared to be more prominent. It is important to note that Figure 2 serves as a qualitative illustration of the shifting ROS profiles; the quantitative data showing the distribution and variance of total ROS and the statistical support for the significant effect of temperature are presented in Figure 1.

A key observation emerged when analyzing the normalized data (Figure 2). At 12°C and 20°C, the relative signal from CellROX Red, which detects superoxide and hydroxyl radicals, surpassed that of the broader‐spectrum CellROX Orange probe. As this is a mathematical consequence of normalizing two probes with different baseline signals and sensitivities, we further explored the biological meaning of this signal crossover.

### 3.3 Principal Component Analysis of ROS Associated Genes Under Different Temperature Conditions

**FIGURE 3:**
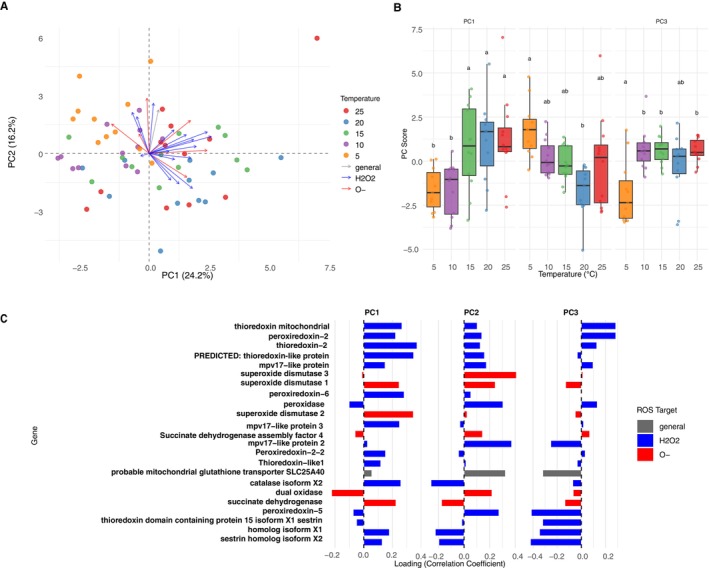
PCA of antioxidant gene expression across five temperatures, showing temperature‐specific patterns and the contribution of ROS‐targeted genes to variation along principal components. (A) Biplot of the first two principal components derived from a principal component analysis (PCA) of 23 antioxidant gene expression profiles across five temperature treatments (5°C, 10°C, 15°C, 20°C, 25°C). Samples are color coded by temperature, and vectors indicate each gene's contribution to the observed variation along PC1 and PC2; the direction of the arrow indicates the temperature range where that gene's expression is higher. Genes are labeled and colored according to their primary ROS target, with gray for general (nonspecific) antioxidants, blue for hydrogen peroxide‐related antioxidants, and red for superoxide‐related antioxidants. (B) Boxplots of sample scores for PC1, PC2, and PC3 across all temperature treatments, illustrating shifts in overall gene expression patterns. Letters indicate statistically significant differences among temperatures within each principal component (Tukey's HSD test, *p* < 0.05) (Data S1). (C) Contribution of 23 ROS production or scavenging candidate genes to PC1, PC2, and PC3. Genes are again categorized by primary ROS target, using the same color scheme as in panel A.

To investigate the overall transcriptional response to temperature, we first analyzed the gene expression profiles from our 58 individual larva samples (11–12 biological replicates per temperature treatment). Principal component analysis (PCA) was performed on normalized read counts of 23 antioxidant genes directly related to either ROS production or scavenging to determine temperature‐dependent expression patterns. Significant PCs were revealed using the Broken Stick model (Data S1), where the first three principal components (PCs) capture different aspects of gene expression variation, with PC1, PC2, and PC3 explaining 24.2%, 16.2%, and 10.8%, respectively. Together, they explained more than 50% of the data (Figure 3A).

The PCA biplot showed a clear temperature‐dependent clustering of the samples. The low‐temperature samples (5°C–10°C) formed distinct clusters in the negative PC1 region, while the high‐temperature samples (20°C–25°C) clustered in the positive PC1 region. Intermediate temperature samples (15°C) occupied transitional positions between these clusters, indicating a gradual shift in gene expression patterns across the temperature gradient.

Analysis of PC scores across temperatures revealed systematic variation in gene expression patterns. PC1 scores showed a strong correlation with temperature, transitioning from negative values at low temperatures to positive values at high temperatures, indicating that genes with positive loadings on PC1 are increasingly expressed at higher temperatures, and vice versa. PC2 scores showed another clear distinction between low temperature (5°C) and high temperature (20°C–25°C), while the 10°C and 15°C data sets fell in the middle. This suggests a threshold of regulation in the antioxidant response system. PC3 provided additional information on cold‐temperature‐specific responses, particularly in the 5°C data (Figure 3B; Data S1).

### 3.4 Differential Gene Expression Highlights a Strong Cold‐Stress Response

To investigate the expression changes of individual genes, we compared each temperature treatment to the 15°C baseline. Genes were considered significantly differentially expressed if they had a False Discovery Rate (FDR) *p* value < 0.05. This analysis revealed a starkly asymmetric transcriptional response to thermal stress (Figure S1). The most profound and widespread changes occurred at the cold extreme (5°C), where numerous antioxidant genes were significantly up‐ or downregulated, indicating a large‐scale transcriptomic reprogramming. In striking contrast, the responses to milder cold (10°C) and warm temperatures (20°C and 25°C) were far more subdued at the individual gene level. Very few genes passed the stringent threshold for statistical significance, suggesting a different regulatory mechanism is at play in response to moderate versus extreme cold stress.

### 3.5 Gene Contributions to Temperature Response

Based on the PCA (Figure 3A), gene loadings were obtained to determine the contribution of the 23 antioxidant genes directly related to either ROS production or scavenging to the temperature response. In PC1, *thioredoxin‐2*, two *mitochondrial thioredoxin isoforms*, two *sup*eroxide *dismutase* variants, *peroxiredoxin‐6*, *catalase isoform X2*, and *mpv17‐like protein 3* showed strong positive loadings (0.2–0.4), indicating higher expression levels at elevated temperatures. In contrast, *dual oxidase* exhibited a negative loading, suggesting increased expression at lower temperatures.

PC2 and PC3 captured variation associated with cold responses. Strong positive loadings in PC2 were observed for *superoxide dismutases, mpv17‐like protein 2, mitochondrial glutathione peroxidase, and peroxiredoxin‐5*, indicating their greater contribution under cold conditions. Negative PC2 loadings for *catalase isoform X2* and *sestrin homologs* suggested differential antioxidant activation at intermediate temperatures. PC3 was dominated by high negative loadings from *sestrin isoforms*, *peroxiredoxin‐5*, and *thioredoxin domain‐containing protein 15*, indicating a specific role for hydrogen peroxide detoxification at low temperatures (Figure 3C; Data S1).

## 4 Discussion

Organisms need to be able to cope with a variety of different temperatures across different time scales and magnitudes. Even though water bodies may buffer temperature fluctuations to a certain extent, in water bodies of temperate regions, and especially small water bodies, temperatures may change on a seasonal, daily, and even minute time scale. Thus, organisms inhabiting these regions need to be adapted to cope with varying conditions, but they may still be vulnerable to temperature extremes. Temperature extremes can exert strong stress on organisms; however, the detection and quantification of this stress are not always easy. This study presents the first direct in vivo measurements of ROS under a broad range of temperatures in an insect. In contrast to earlier studies that employed indirect methods, such as enzyme activity assays or in vitro ROS measurements (Cui et al. 2011; Jia et al. 2011; Yang et al. 2010; Zhang et al. 2015), our direct in vivo measurements of ROS provide a more comprehensive and precise understanding of ROS production in response to a range of temperatures encountered by the species in their natural habitat. While acknowledging the theoretical potential for CellROX compounds to induce minor background oxidative responses, our optimized nonlethal concentrations and consistent application across all treatments ensured that the observed temperature‐dependent differences accurately reflected biological variation rather than probe‐induced artifacts.

### 4.1 Temperature‐Dependent Stress Response

Our findings demonstrated that ROS levels in *C. riparius* followed a U‐shaped pattern across the temperature gradient, with potentially elevated oxidative stress at both upper and lower temperature extremes. The increased ROS levels at the extreme temperatures aligned with observations in other ectotherms, where temperature extremes challenge cellular homeostasis. For instance, in ectothermic species like fish and amphibians, either heat or cold stress can lead to increased ROS (Paital and Chainy 2014).

In ectotherms at high temperatures, the metabolic rate increases, leading to higher oxygen consumption and elevated ROS production as a metabolic by‐product (Rollins‐Smith and Le Sage 2023). Consistent with the generally increased metabolic rate observed at higher temperatures (Holden et al. 2022; Payne et al. 2025), our transcriptomic analysis identified a linearly increasing expression pattern for five *NADH dehydrogenase* genes and an exponential increase for a *cytochrome c reductase* gene with increasing temperature, which is consistent with enhanced expression of components within complexes I and III of the electron transport chain. This pattern likely reflected a response to elevated energy demands. As temperatures rise, cellular ATP requirements increase, driving the need for efficient mitochondrial ATP production (Twardochleb et al. 2023). The observed increasing pattern in mitochondrial gene transcription may therefore serve to increase the flow of electrons through the electron transport chain to meet these heightened energy demands under thermal stress (Park and Kwak 2014).

On the other hand, at lower temperatures, mitochondrial function is disrupted as enzyme function slows down and oxygen consumption is decreased, leading to increased electron leakage from the mitochondrial electron transport chain and subsequent enhancement of ROS production (Jørgensen et al. 2023). In accordance with this, we observed a general decreasing pattern of complex I and III genes at lower temperatures, which may represent a compensatory metabolic adjustment rather than dysfunction, aligning with diminished energy requirements in cold conditions. This decreasing trend of the specific genes in cold conditions might be a strategy to conserve energy and minimize ROS production specifically from the electron transport chain's activity, which is particularly useful in low‐temperature environments where ATP demand decreases (Colinet et al. 2017).

While our direct in vivo ROS measurements provided an unprecedented view of real‐time oxidative activity, future studies should complement these findings by quantifying specific oxidative damage biomarkers to fully assess the consequences of this stress.

### 4.2 Different ROS Profiles Across the Temperature Gradient

Beyond the observed U‐shaped stress response, our approach of using different CellROX reagents further disentangled these responses: at low temperatures, the superoxide group (superoxide and hydroxyl radical) appears to predominate, whereas at high temperatures, the hydrogen peroxide group (hydrogen peroxide, peroxynitrite anion, and nitric oxide) suggests a greater contribution.

We propose that the signal crossover at 12°C and 20°C, where the normalized CellROX Red signal surpassed the Orange, reflects a significant, temperature‐specific shift in the ROS composition. At 20°C, a temperature supporting high metabolic activity, the targeted production of superoxide is a critical component of cellular signaling pathways (Averill‐Bates 2023; Sies and Jones 2020). The elevated CellROX Red signal here is powerfully substantiated by our transcriptomic data, which reveal that expression loadings for key superoxide‐scavenging genes are significantly correlated with the higher Red signal at this exact temperature (Park et al. 2012). This indicates a direct biological response: the cell is actively enhancing its defense machinery to manage a higher, physiologically relevant flux of superoxide. Conversely, the crossover at the mild cold‐stress temperature of 12°C likely results from altered mitochondrial electron transport efficiency, leading to increased superoxide leakage (Zhao et al. 2019). Therefore, we interpret this signal crossover not as a methodological artifact, but as a key finding that distinguishes between two distinct modes of ROS production: a managed flux for cellular signaling near optimal temperatures and mitochondrial leakage in response to cold.

At low temperatures (e.g., 4°C), the elevated ROS production was largely driven by hypoxia‐induced hemoglobin autoxidation. In *C. riparius* larvae, hemoglobin levels are known to be high and increase further under hypoxic conditions (Choi et al. 2000). Under such low‐oxygen environments, reduced oxygen diffusion promotes autoxidation of oxyhemoglobin to methemoglobin (Lushchak 2011), a process that generates superoxide radicals and, to a lesser extent, hydrogen peroxide (Yan et al. 2020). Furthermore, diminished peroxidase activity under these hypoxic conditions favors the Fenton reaction, thereby forming hydroxyl radicals. Such molecular mechanisms not only explain the ROS profile observed at low temperatures but may also account for the associated developmental delays observed below 15°C (Alter et al. 2024).

At elevated temperatures, the predominance of the hydrogen peroxide group aligns directly with our transcriptomic findings. We observed a general trend of temperature‐dependent upregulation for genes encoding key components of the mitochondrial electron transport chain. This transcriptional response suggests an increased capacity for mitochondrial respiration, which would elevate the rate of electron flow and, consequently, the leakage of superoxide anions from the ETC (Messina et al. 2023). Upregulated *superoxide dismutase* activity then converts these anions into hydrogen peroxide (Murphy 2009). Beyond this general metabolic response, heat stress triggers additional molecular responses such as increased nitric oxide production through the modulation of *nitric oxide synthase* that promote the formation of peroxynitrite when superoxide reacts with nitric oxide (Piacenza et al. 2022; Figure S3).

The observed molecular processes reflect the knowledge about the ecology of *C. riparius* populations. Elevated temperatures accelerate larval development, thereby reducing generation time, which principally increases fitness (Foucault et al. 2019). However, the trade‐offs are significant: increasing temperatures increasingly compromise larval survival, reduce pupation success, and thus overall impair adult reproductive fitness (Foucault et al. 2018; Nemec et al. 2013). Experiments have shown that while optimal growth and relatively short generation times are maintained between 16°C and 23°C (Oppold et al. 2016), exposure to temperatures around 28°C leads to a notable decline in population growth (Nemec et al. 2013). This suggests that the benefits of accelerated growth rates at elevated temperatures are counterbalanced, among others, by the physiological costs of the increased ROS production shown here.

Our findings may also have evolutionary implications for the species. The mutation rate response across temperatures reported by Waldvogel and Pfenninger (2021) exhibited an optimality pattern, superficially similar to the one observed in our ROS measurements. Observed mutation rates had their minimum at ~18°C and increased with increasing temperatures, but also towards colder temperatures, reaching a maximum at 12°C (Waldvogel and Pfenninger 2021). Below this temperature, reproduction is not possible anymore in *C. riparius*, and therefore, transgenerational mutation rates could not be measured. However, the current study has shown that ROS levels had their minimum at 12°C and therefore may not be the sole or primary driver for the increased mutation rate observed at this temperature. One plausible hypothesis is that the extended generation times at cold temperatures (Oppold et al. 2016) allow even low ROS levels or other mechanisms more time to inflict DNA damage (Beal et al. 2019) and thus increase mutation rates. However, the overwintering generation of *C. riparius* in nature is exposed for several months to much lower temperatures down to 4°C (Pfenninger et al. 2023). The increased ROS level at these cold temperatures observed here could thus still be a major driver of mutagenesis in natural populations, due to the interplay of increased oxidative cold stress and extended exposure time during hibernation.

It is crucial to interpret these findings in the context of our study design, which utilized a single laboratory population originating from a temperate climate in Germany. Given the broad geographical distribution of *C. riparius* across diverse climatic regimes, from the Mediterranean to Northern Europe, local adaptation in thermal physiology is highly probable (Waldvogel et al. 2018). Therefore, the specific thermal optima and stress thresholds we report here are certainly not universal for the species. This underscores that population‐specific variation is a critical factor, and our study provides a foundational baseline for future comparative population studies aimed at dissecting the genetic basis of local thermal adaptation.

Furthermore, the responses documented in this study represent the acute plastic response to a novel thermal environment, as all larvae were reared at a constant 20°C. It is well established that an organism's thermal history can profoundly shape its physiological and molecular responses to stress. For example, acclimation history, through which an individual gradually adjusts to a new thermal regime, can lead to the restructuring of metabolic pathways or the accumulation of protective molecules, potentially dampening the acute ROS production we observed upon a sudden temperature shift (Shama et al. 2014). Similarly, transgenerational plasticity, often mediated by maternal effects, can preprogram offspring for the environment their parents experienced (Bonzi et al. 2025). A mother exposed to colder conditions might provision its eggs with specific antioxidants or transcripts that alter the baseline stress response of its larval offspring, leading to a different oxidative stress profile than the one we measured (Marks and Lailvaux 2024). Therefore, while our results provide a crucial baseline for understanding the immediate mechanisms of thermal stress, they should be interpreted as one component of the broader thermal biology of *C. riparius* .

### 4.3 Molecular Mechanisms of Antioxidant Defense Across Temperature Gradients

Our RNA‐Seq approach, which analyzed individual larvae, allowed us to capture interindividual variation in gene expression, which is crucial for understanding the full spectrum of plastic responses and genetic variation within a population (Pfenninger et al. 2025). This analysis revealed a targeted gene expression response by which *C. riparius* reacted to temperature‐induced oxidative stress, corroborating the physiological level results observed above. At higher temperatures (20°C–25°C), our analysis revealed a response favoring the management of hydrogen peroxide. Our transcriptomic data provide a direct mechanistic basis for this observation. We saw a general trend of upregulation for genes encoding key components of the mitochondrial electron transport chain (ETC), such as *NADH dehydrogenases* (Complex I) and *cytochrome c reductase* (Complex III). This suggests an increased metabolic rate and electron flow to meet higher energy demands, which inevitably increases the leakage of superoxide from the ETC (Murphy 2009). The cellular response to this primary challenge was evident in the concurrent positive contribution of *superoxide dismutase* variants, whose function is to convert this excess superoxide into hydrogen peroxide. Consequently, the system then activated defenses to manage this secondary product, as shown by the strong contribution of hydrogen peroxide‐neutralizing enzymes like *thioredoxins*, *peroxiredoxin‐6*, and *catalase isoform X2* to the variance in gene expression at warm temperatures, as revealed by our PCA (Figure 3C). Thus, the ROS profile at high temperatures appears to be a direct consequence of an elevated metabolic rate (Pamenter et al. 2018), followed by a coordinated, two‐step enzymatic defense. However, it is important to note that our transcriptomic analysis did not include the 28°C treatment, which elicited the highest physiological ROS production. Future studies incorporating this upper thermal limit would be valuable for elucidating the transcriptional signatures associated with the transition from adaptive response to acute heat stress.

Conversely, the response to colder conditions (5°C–10°C) was mechanistically distinct. Physiologically, these temperatures were dominated by superoxide anion oxidant, a pattern that cannot be explained by an elevated metabolic rate. Our transcriptomic analysis, however, provides a compelling molecular explanation. The transcriptional reprogramming at cold temperatures was the most pronounced observed across the entire gradient, with a large number of genes being significantly differentially expressed at 5°C (Figure S1). Central to this response was the significant upregulation of *dual oxidase*, a membrane‐bound enzyme known to be a potent producer of superoxide (Donkó et al. 2005). This specific gene induction offers a direct molecular source for the observed superoxide surge, fitting the physiological data perfectly. Furthermore the antioxidant system, in turn, mounted a complex defense tailored to this specific threat. The primary challenge of superoxide under cold temperatures, was met with a complex regulation of *superoxide dismutase* variants (Figure 3C), whose role is to convert superoxide to the less reactive hydrogen peroxide. However, this enzymatic conversion creates a secondary oxidative challenge. Accordingly, the cold response also involved the regulation of hydrogen peroxide scavengers, as indicated by the strong contribution of genes like *peroxiredoxin‐5* and *mpv17‐like protein 2* to the variance observed in the PCA at low temperatures (Figure 3C). This reveals a sophisticated, multi‐step defensive strategy. The cold‐temperature response, therefore, appropriately emphasizes primary superoxide management, but also prepares for its secondary byproducts. This seemingly counterintuitive production and management of multiple ROS species at low temperatures may serve critical signaling functions, potentially activating cold‐adaptive pathways or maintaining cellular redox homeostasis despite reduced metabolic activity (Jørgensen et al. 2023; Murphy 2009).

## 5 Conclusion and Future Directions

This study provides the first direct in vivo evidence for a U‐shaped pattern of oxidative stress across a broad temperature range in the insect *C. riparius* , with minimal ROS levels observed within a thermal window of 12°C–18°C. We demonstrate that this pattern arises from mechanistically distinct challenges: superoxide predominance at low temperatures, linked to hemoglobin autoxidation, and hydrogen peroxide predominance at high temperatures, driven by increased metabolic rate. Correspondingly, the transcriptional response of antioxidant genes is tailored to counter the specific ROS type prevalent at each thermal extreme. These findings establish that temperature extremes impose distinct forms of oxidative stress, a mechanism that plausibly impacts organismal fitness and likely influences the ecology and evolution of the species by exerting complex selective pressures. While our results provide a crucial mechanistic baseline, future work is needed to explore how these responses are modulated by factors such as local adaptation, as our findings are based on a single population. Further studies should also investigate the transcriptional tipping point at extreme high temperatures and how long‐term acclimation and developmental plasticity alter these acute stress responses. Such research will be critical for predicting the vulnerability and adaptive potential of freshwater ectotherms in a changing world.

## Author Contributions

**Burak Bulut:** conceptualization (equal), data curation (equal), formal analysis (equal), methodology (equal), visualization (equal), writing – original draft (equal). **Maximilian Geiss:** conceptualization (equal), data curation (equal), formal analysis (equal). **Markus Bernard:** methodology (equal). **Halina Binde Doria:** conceptualization (equal), methodology (equal). **Barbara Feldmeyer:** conceptualization (equal), data curation (equal), writing – review and editing (equal). **Markus Pfenninger:** conceptualization (equal), funding acquisition (equal), methodology (equal), project administration (equal), resources (equal), validation (equal), writing – review and editing (equal).

## Funding

This work was supported by Deutsche Forschungsgemeinschaft, PF 390/15‐1.

## Conflicts of Interest

The authors declare no conflicts of interest.

## Supporting information


**Data S1:** ece372625‐sup‐0001‐DataS1.xlsx.

**Data S2:** ece372625‐sup‐0002‐Appendix.docx.

## Data Availability

The data is accessible at the European Nucleotide Archive (ENA) under accession number PRJEB89193. All other primary data supporting the results of this study, including raw fluorescence intensity measurements and sample metadata, can be found at Zenodo doi: https://doi.org/10.5281/zenodo.17184450.
