# Supplementary material for: Dissecting Oxidative Stress and Organismic Response to a Temperature Gradient in the Midge Chironomus riparius
Source: Ecol Evol. 2025 Dec 11;15(12):e72625. doi: 10.1002/ece3.72625 (PMC12698206; doi:10.1002/ece3.72625)

**Appendices**

**Figure S1: Volcano plots of differential gene expression for ROS-related genes.** Each plot compares a thermal treatment against the 15°C baseline: (A) 5°C, (B) 10°C, (C) 20°C, and (D) 25°C. The x-axis represents the log2 fold change in expression, and the y-axis represents the statistical significance (-log10 adjusted p-value). Genes are colored by their primary ROS target.

**
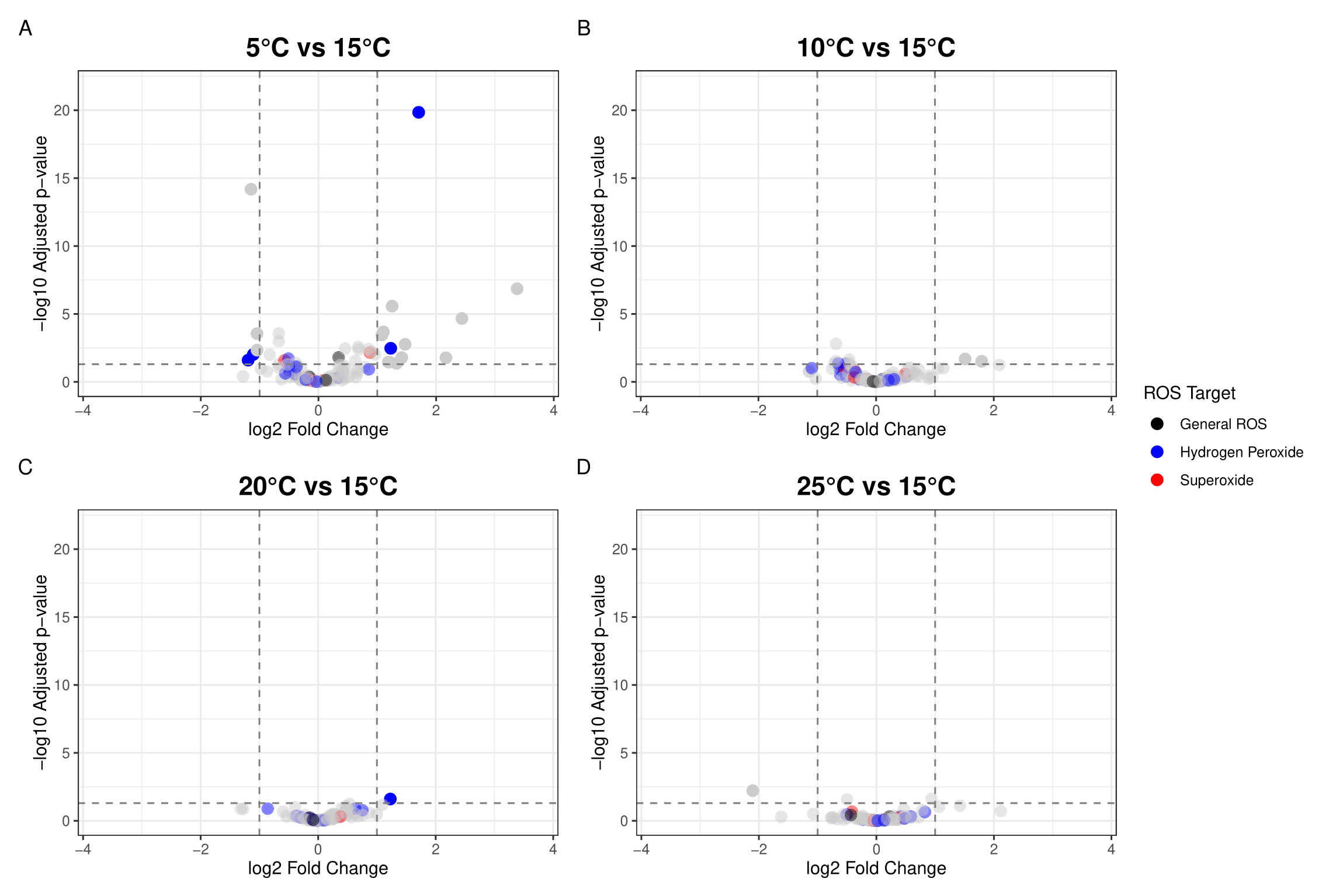
**

**Figure S2: Representative images of the larval segment used for fluorescence measurements.** The images show the first abdominal segment of a *Chironomus riparius* L3 stage larva under fluorescence (left column) and brightfield (right column) microscopy, illustrating the standardized location for quantifying relative fluorescence intensity (RFI).


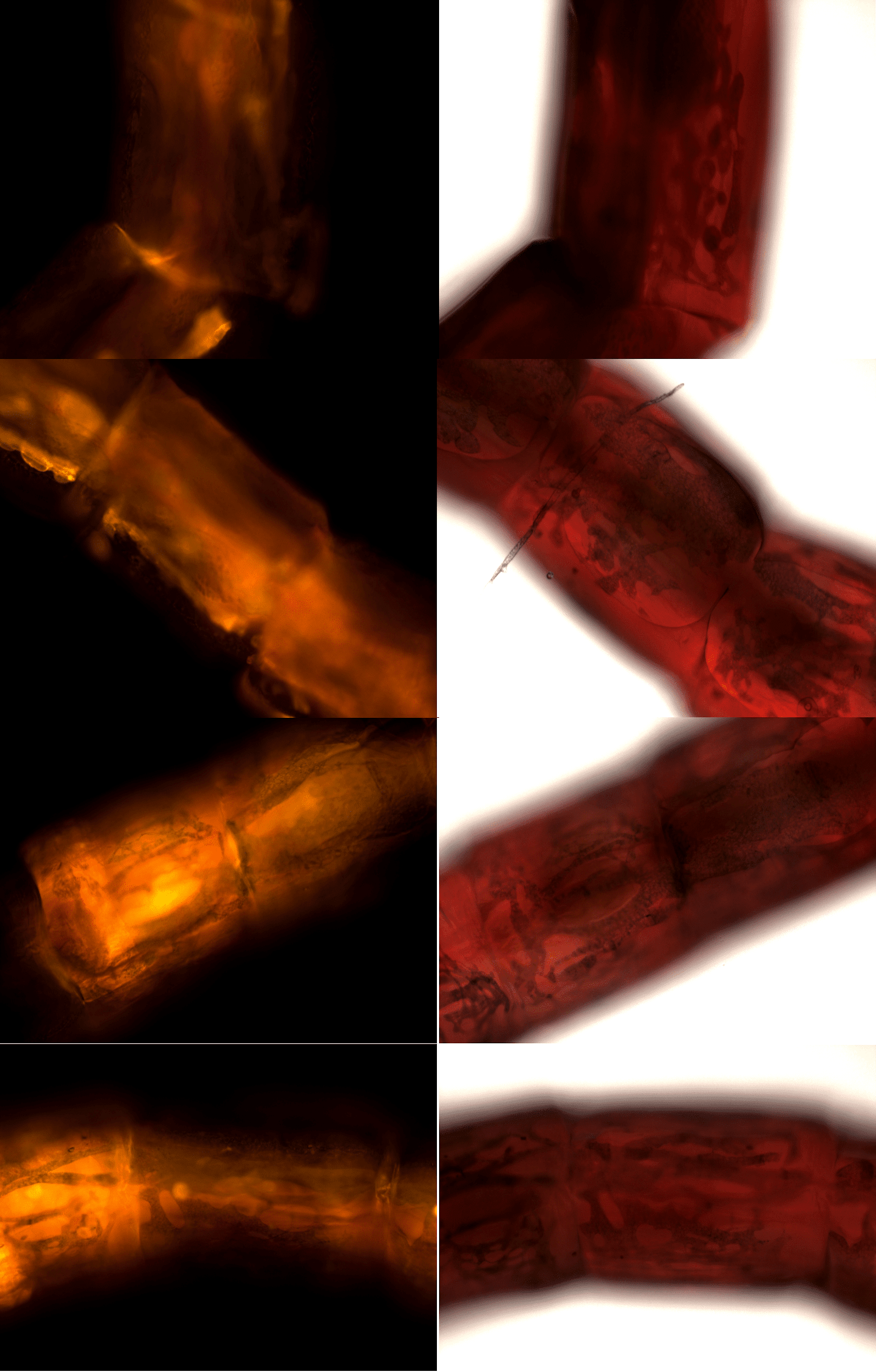


**Figure S3:** **Illustration of the mitochondrial-originated conversion pathways of reactive oxygen species (ROS) in animals, highlighting specific ROS detected by CellROX Red and CellROX Orange.** In animals, the primary ROS produced is superoxide from mitochondria, which is then converted to a range of different ROS. While a part is converted to hydrogen peroxide by superoxide dismutase, others may be transformed to peroxynitrite in the presence of nitric oxide radicals, which are mainly produced by nitric oxide synthase family enzyme, which can react with superoxide to form peroxynitrite anion (Halliwell et al., 1999). Additionally, hydrogen peroxide may be converted in an additional step to a hydroxyl radical through the Fenton reaction by the endoplasmic reticulum (Liu et al., 2004; Sun et al., 2018; Temple et al., 2005). Red-labeled species indicate oxidants specifically detectable by CellROX Red (e.g., superoxide and hydroxyl radical), while orange-labeled species indicate those uniquely detectable by CellROX Orange (e.g., hydrogen peroxide, peroxynitrite, and nitric oxide).


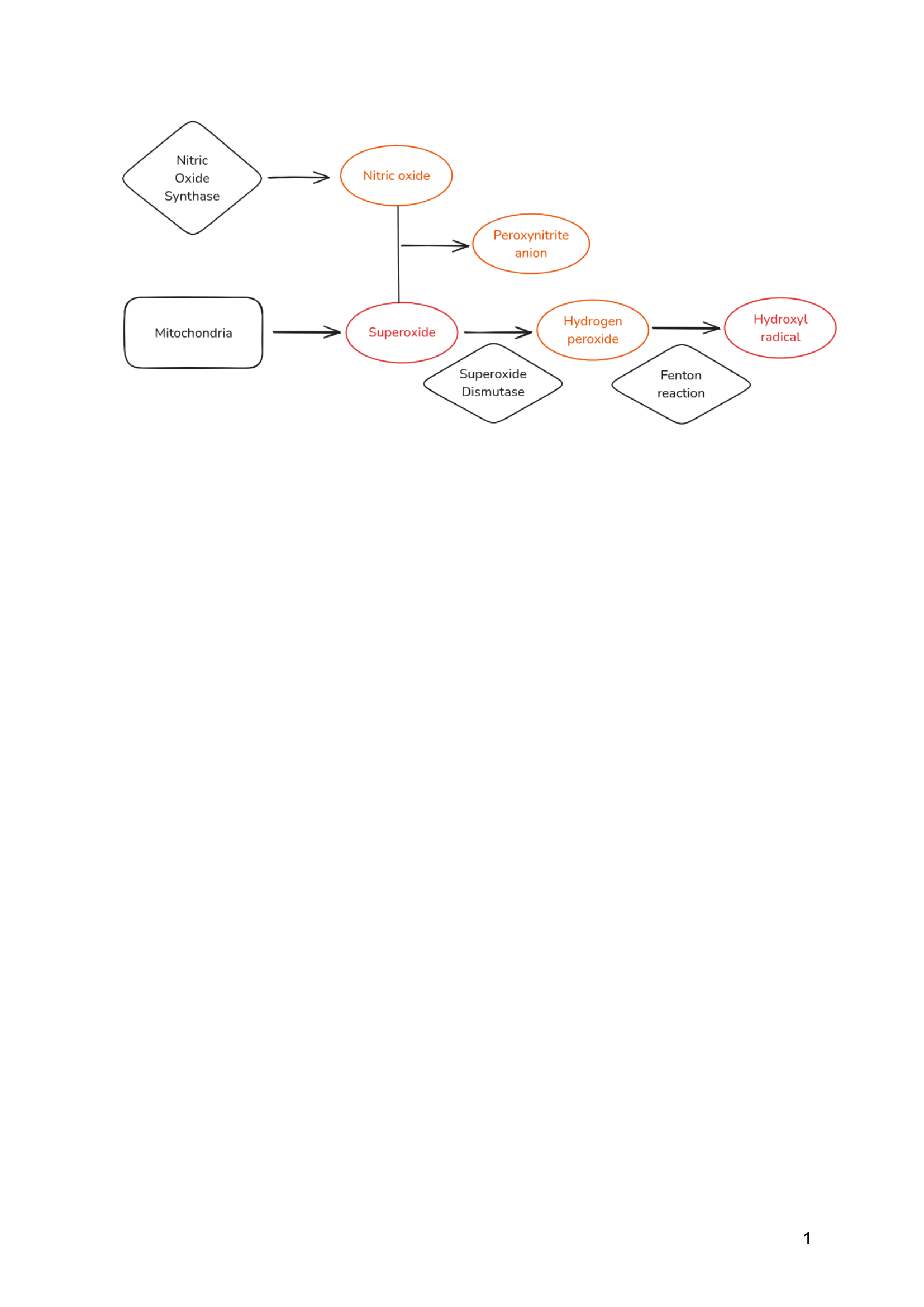

Supplement: Supplementary file 2 — Data S2: ece372625‐sup‐0002‐Appendix.docx. [file ECE3-15-e72625-s001.docx]
